# Supplementary material for: Modeling development of tertiary lymphoid structures in pulmonary tuberculosis by 3D profiling and trajectory analysis
Source: bioRxiv. 2026 Jun 18:2026.06.02.725201. Originally published 2026 Jun 7. Preprint. [Version 2] doi: 10.64898/2026.06.02.725201 (PMC13252128; doi:10.64898/2026.06.02.725201)
Supplement: Supplementary file 1 [file NIHPP2026.06.02.725201v2-supplement-5.pdf]

bioRxiv preprint doi: https://doi.org/10.64898/2026.06.02.725201; this version posted June 18, 2026. The copyright holder for this preprint (which was not certified by peer review) is the author/funder, who has granted bioRxiv a license to display the preprint in perpetuity. It is made available under aCC-BY 4.0 International license.

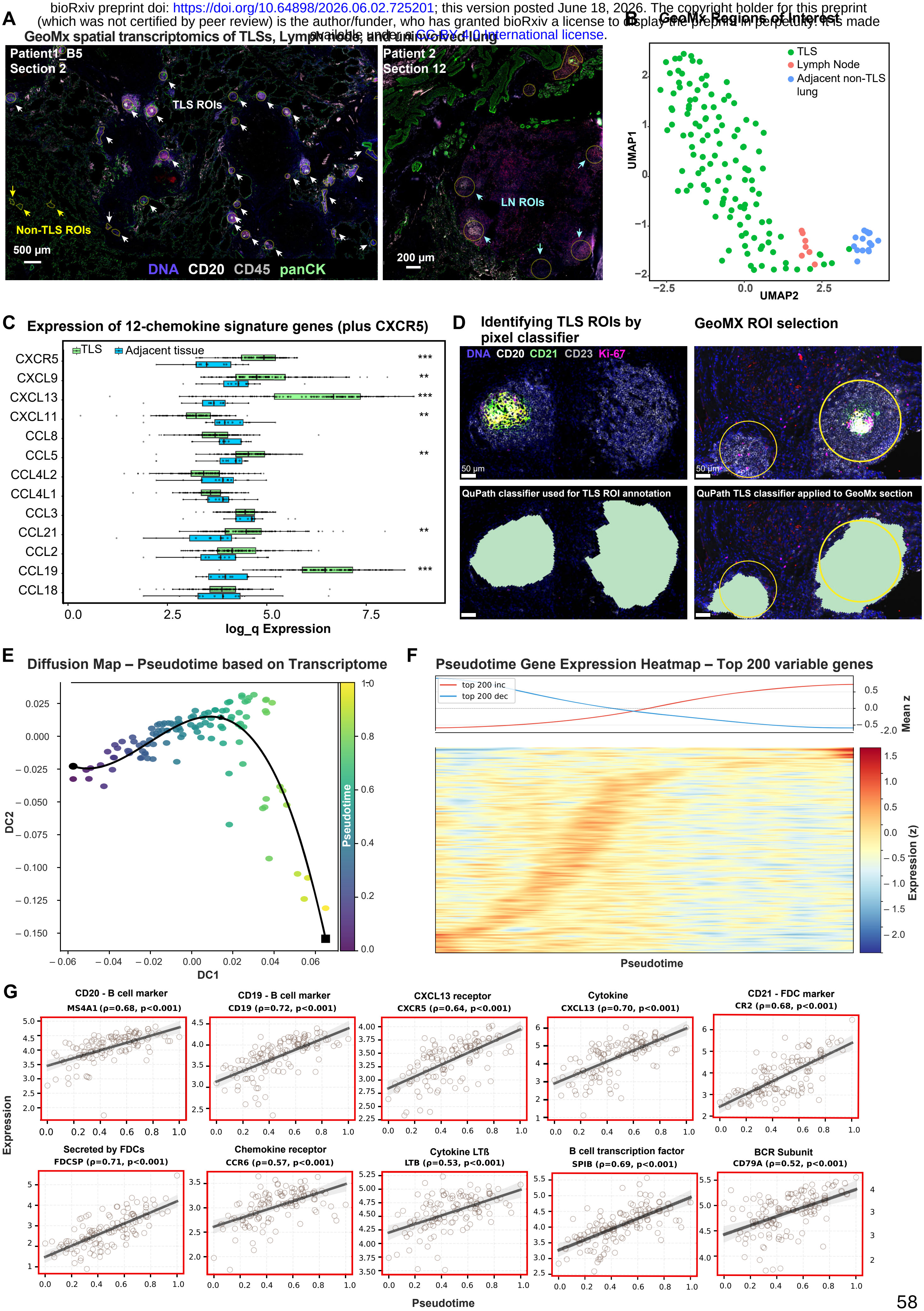

bioRxiv preprint doi: https://doi.org/10.64898/2026.06.02.725201; this version posted June 18, 2026. The copyright holder for this preprint (which was not certified by peer review) is the author/funder, who has granted bioRxiv a license to display the preprint in perpetuity. It is made available under a CC-BY 4.0 International license.

Figure S1. Spatial transcriptomic validation of TLS identity in TB-infected human lung tissue, related to Figure 1

(A) GeoMx spatial transcriptomics of tertiary lymphoid structures (TLSs), lymph node, and uninvolved lung alveoli from TB-infected human lung samples. Representative regions of interest (ROIs) are shown from Patient1_B5 (section 2) and Patient2 (section 12), overlaid on immunofluorescence images (DNA, CD20, CD45, pan-cytokeratin).

(B) UMAP embedding of GeoMx ROIs colored by histological region, demonstrating transcriptional separation between lymphoid structures (TLSs, lymph node) and uninvolved lung regions.

(C) Expression of members of the 12-chemokine (12-CK) TLS gene signature, together with CXCR5, in TLSs compared to uninvolved lung (UL) regions. Boxplots show log_2-transformed Q3-normalized expression values for individual regions of interest, with TLS regions (green) and UL regions (light blue). Boxes represent the interquartile range (25th–75th percentiles), the center line indicates the median, and points represent individual measurements. CXCR5 is shown for comparison but is not part of the canonical 12-chemokine TLS signature.

(D) Cross-modal validation of TLS identification. Top left: Representative TLS from Patient1_B4 (used for 3D reconstruction) highlighting B-cell and follicular organization. Top right: Multiplexed CyCIF image from Patient1_B5 (section 2; post-GeoMx CyCIF) showing a TLS ROI with the indicated markers (DNA, CD20, CD21, CD23, Ki-67). Yellow outlines indicate annotated GeoMx TLS boundaries. Bottom panels: Application of a QuPath TLS pixel classifier to identify TLS regions in both the 3D reconstruction and GeoMx sections, demonstrating concordant detection across modalities and validating the annotated TLS ROIs in serial section reconstruction experiment as *bona fide* TLSs.

(E) Diffusion map of 2D TLS transcriptional states using GeoMx ROIs, colored by transcriptomic pseudotime. Each point represents one ROI plotted in diffusion space (DC1, DC2). A principal curve (solid black line) indicates the inferred maturation trajectory.

(F) Heatmap of the top 200 most variable genes (ranked by coefficient of variation) across 2D TLSs. Z-scored gene expression (± 2) is plotted along pseudotime. Overlay lines indicate average z-score trends of the top 200 most variable increasing (red) and decreasing (blue) gene sets along the pseudotime.

(G) Signatures of TLS pseudotime based on transcriptome. Log_2-transformed Q3-normalized expression of selected genes along pseudotime. Each panel reports Spearman ρ and p-value.

bioRxiv preprint doi: https://doi.org/10.64898/2026.06.02.725201; this version posted June 18, 2026. The copyright holder for this preprint (which was not certified by peer review) is the author/funder, who has granted bioRxiv a license to display the preprint in perpetuity. It is made available under aCC-BY 4.0 International license.

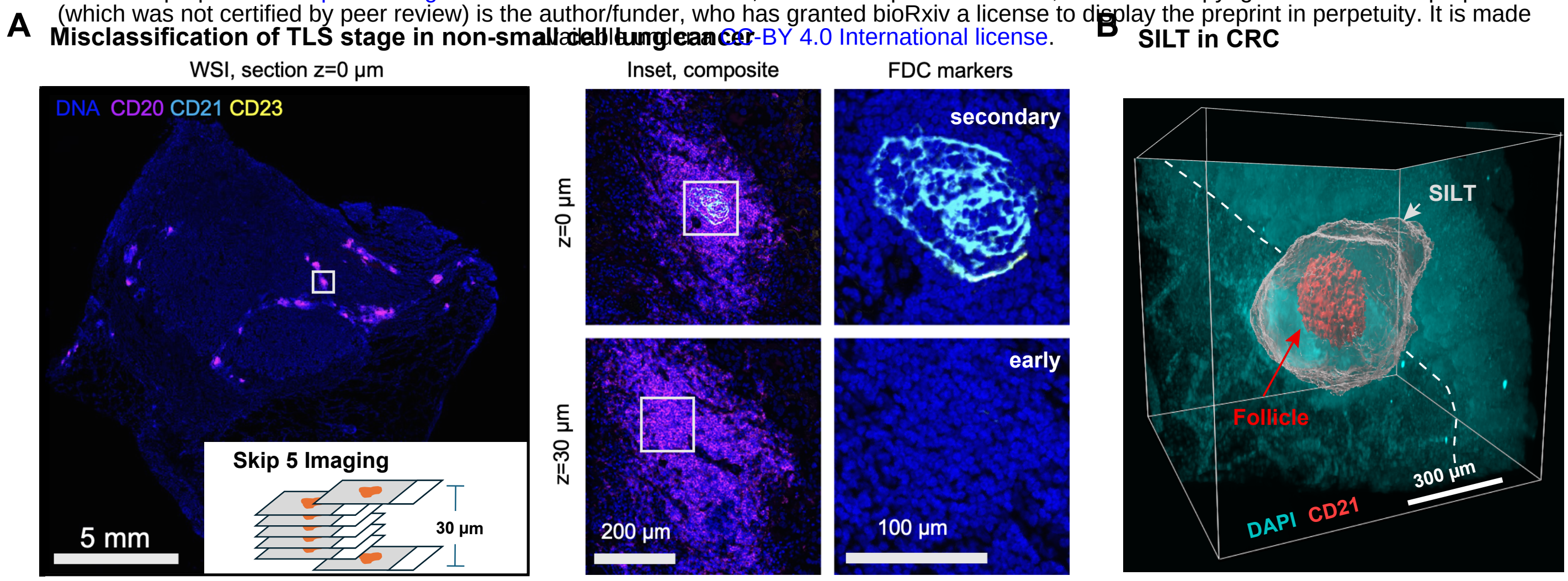

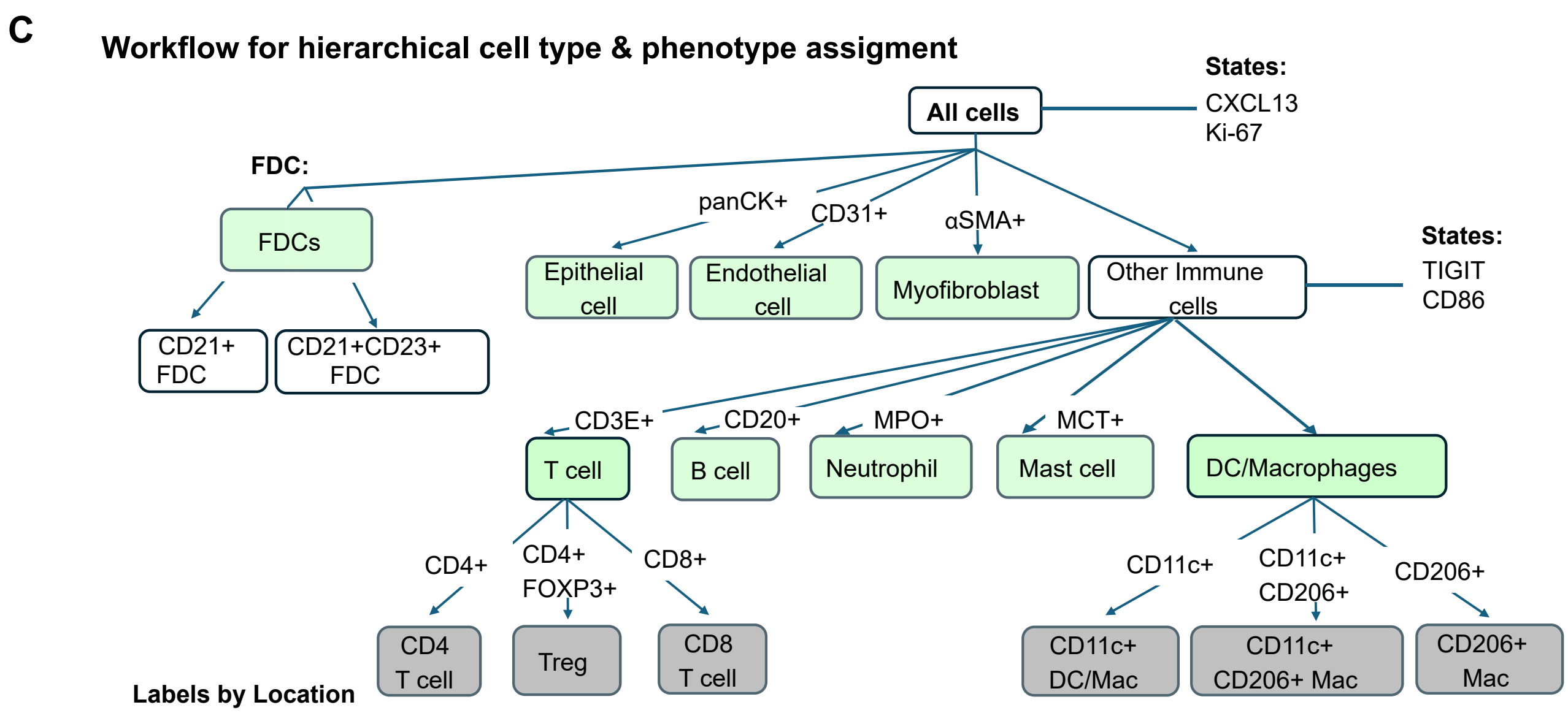

Labels by Location

**Follicular:**			
TIGIT+ :	Tfh	Tfr	Tfc
TIGIT - :	TIGIT - CD4+	TIGIT - Treg	TIGIT - CD8+
**Extrafollicular:**	extrafollicular CD4+	extrafollicular Treg	extrafollicular CD8+

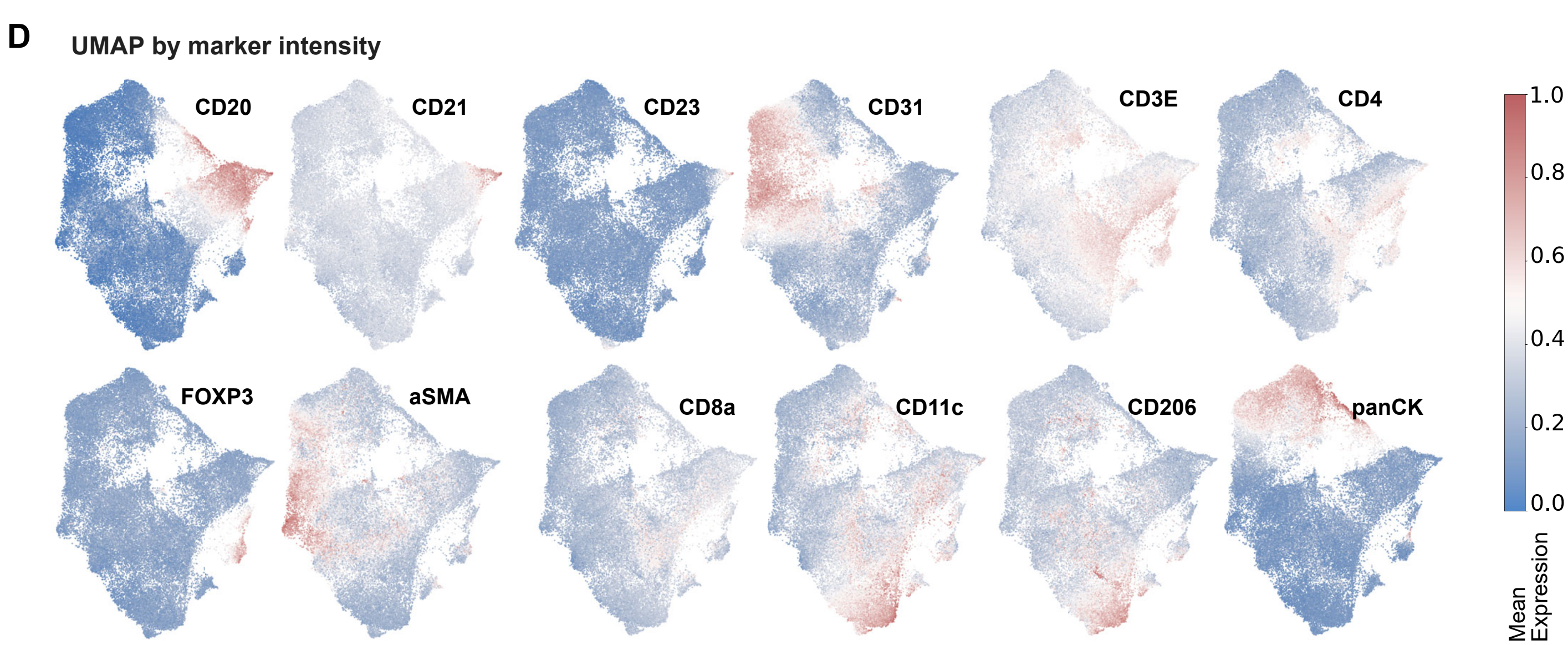

bioRxiv preprint doi: https://doi.org/10.64898/2026.06.02.725201; this version posted June 18, 2026. The copyright holder for this preprint (which was not certified by peer review) is the author/funder, who has granted bioRxiv a license to display the preprint in perpetuity. It is made available under a CC-BY 4.0 International license.

Figure S2. Examples highlighting limitations of 2D TLS classification in other tissues and the cell phenotyping strategy used for TB TLS profiling, related to Figure 1

(A) TLS misclassification observed in non-small cell lung cancer tissue sample.
(B) 3D image of representative solitary intestinal lymphoid tissue (SILT) in colorectal cancer tissue. The $CD21^+$ follicular dendritic cell (FDC) network occupies only ~8.4% of the total SILT volume, highlighting the potential for misclassification in 2D tissue sections if the follicular compartment is not captured.
(C) Phenotyping workflow for assigning cell types.
(D) Uniform manifold approximation and projection (UMAP) plot of single-cell data from CyCIF imaging (Patient1_B4_Section 2), colored by marker intensity. UMAP was generated from randomly sampled 50,000 single cells. See **Figure 1F**.

bioRxiv preprint doi: https://doi.org/10.64898/2026.06.02.725201; this version posted June 18, 2026. The copyright holder for this preprint (which was not certified by peer review) is the author/funder, who has granted bioRxiv a license to display the preprint in perpetuity. It is made available under a CC-BY 4.0 International license.

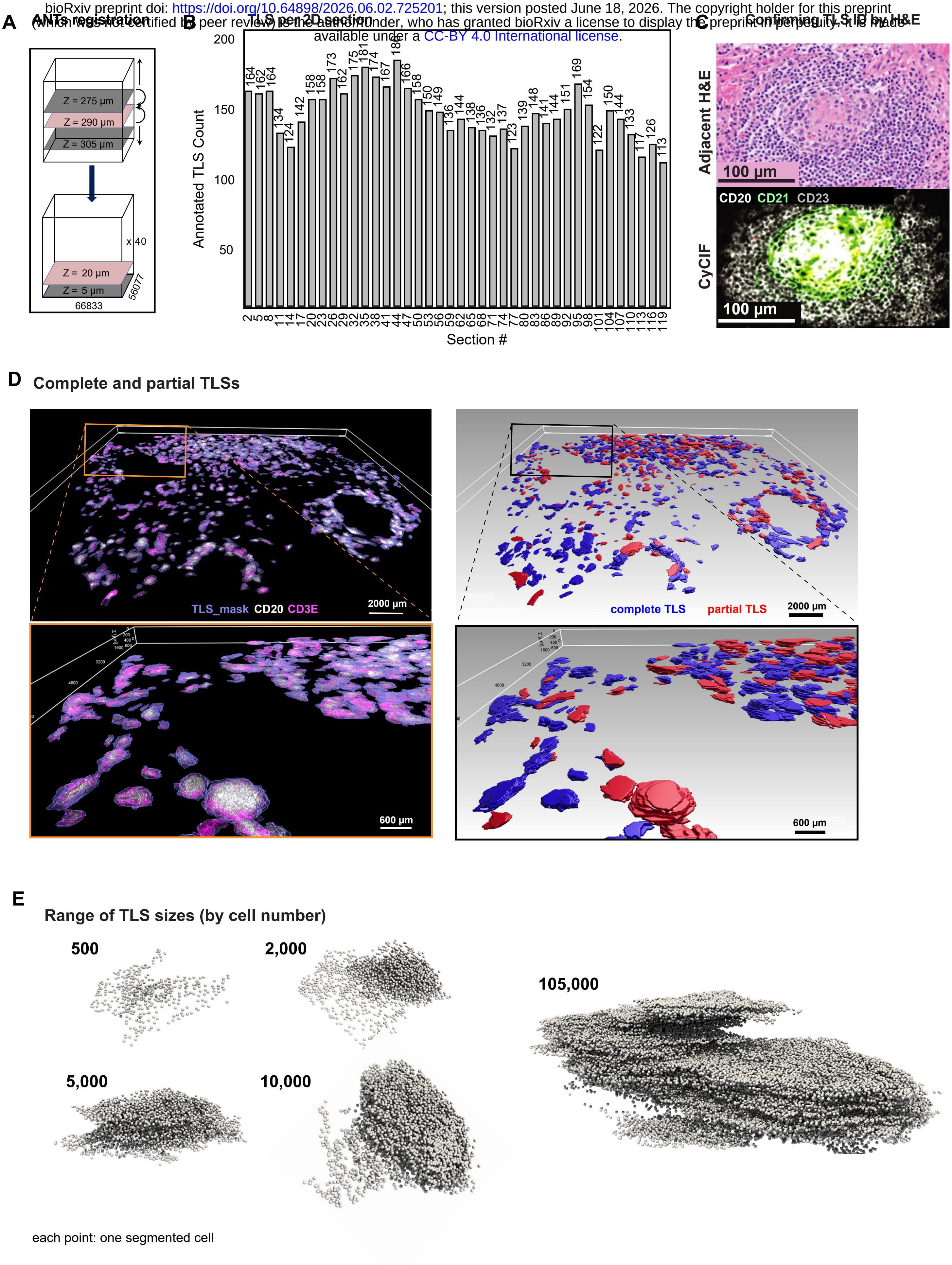

bioRxiv preprint doi: https://doi.org/10.64898/2026.06.02.725201; this version posted June 18, 2026. The copyright holder for this preprint (which was not certified by peer review) is the author/funder, who has granted bioRxiv a license to display the preprint in perpetuity. It is made available under a CC-BY 4.0 International license.

Figure S3. Three-dimensional reconstruction reveals heterogeneous TLS architectures in pulmonary TB, related to Figure 1

(A) Representation of ANTs-based registration pipeline for CyCIF serial section alignment to generate a spatially coherent 3D tissue volume.

(B) Number of annotated TLS regions per serial section across Patient1_B5, illustrating section-to-section variability in TLS detection, motivating volumetric reconstruction.

(C) Representative TLS ROIs showing concordant H&E morphology and CyCIF marker expression. H&E-to-CyCIF registration enabled comparison between annotation of TLSs by both modalities. Scale bars, 100 μm.

(D) Assessment of volumetric completeness of TLSs in TB-infected lung. Right panels: Whole-slide and higher-magnification CyCIF images showing TLS masks (blue) with CD20 (white) and CD3E (magenta) staining. Left panels: Corresponding surface-rendered 3D reconstructions of TLS objects generated from serial sections. TLSs fully contained within the reconstructed image volume were classified as complete (blue), whereas TLSs truncated by volume boundaries were classified as partial or incomplete (red).

(E) Representative 3D renderings of TLSs spanning the size range in dataset, from small aggregates to very large extended structures. Cells are rendered in ParaView.

bioRxiv preprint doi: https://doi.org/10.64898/2026.06.02.725201; this version posted June 18, 2026. The copyright holder for this preprint (which was not certified by peer review) is the author/funder, who has granted bioRxiv a license to display the preprint in perpetuity. It is made available under a CC-BY 4.0 International license.

A

TLS class or subgroup	Frequency
Early TLSs	70
Primary TLSs	77
Secondary TLSs	323
Total TLSs (complete and QC-passed)	470
Secondary TLSs (with Ki-67+ GC cells)	109
Multifocal TLSs (CD21+ or CD21+CD23+)	93
Granuloma-associated TLSs (GrALTs)	293
Unassociated TLSs	177

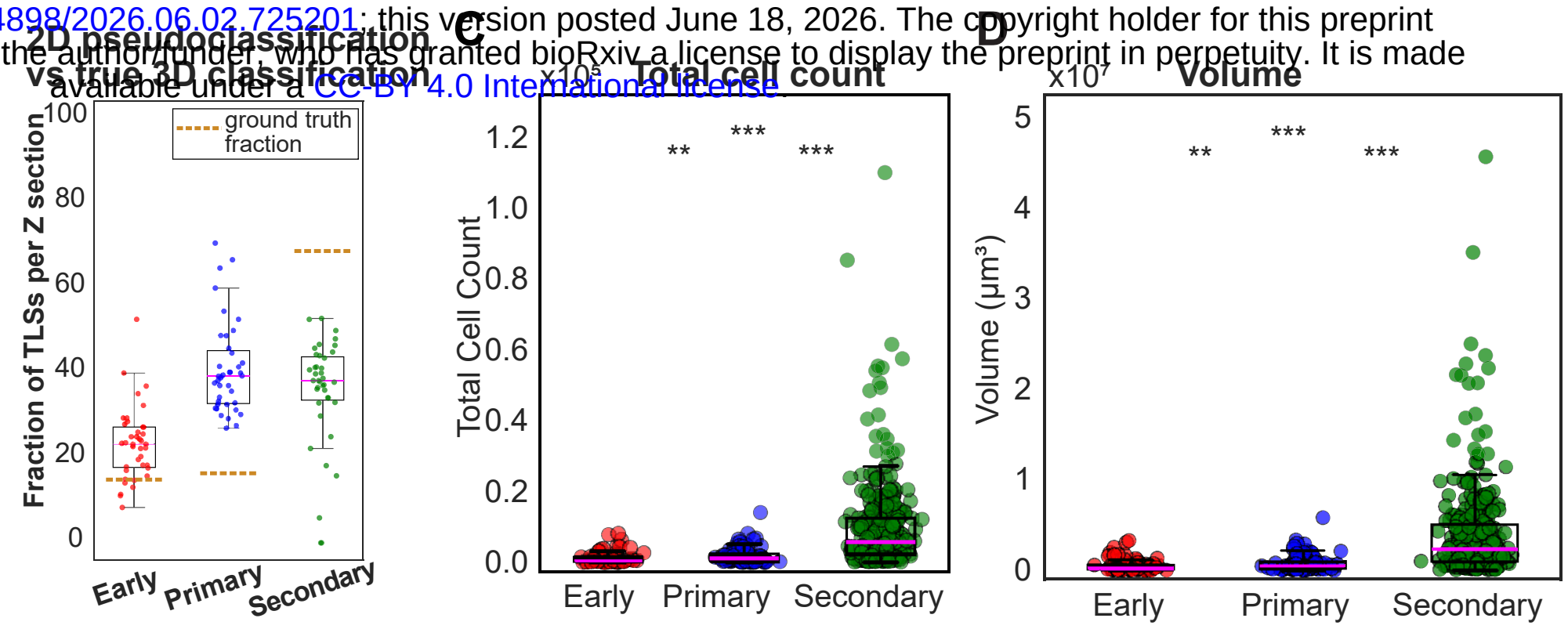

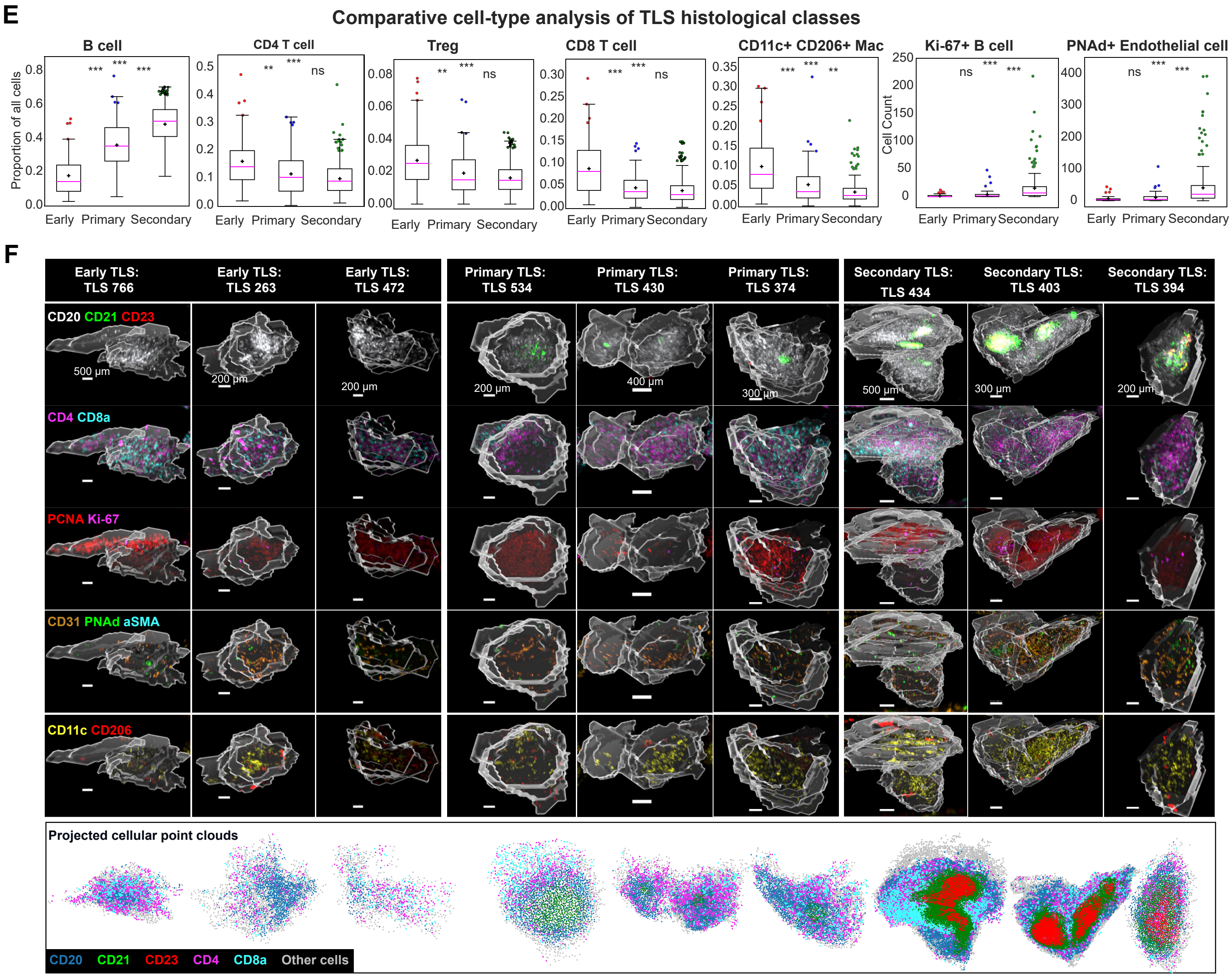

bioRxiv preprint doi: https://doi.org/10.64898/2026.06.02.725201; this version posted June 18, 2026. The copyright holder for this preprint (which was not certified by peer review) is the author/funder, who has granted bioRxiv a license to display the preprint in perpetuity. It is made available under a CC-BY 4.0 International license.

Figure S4. Three-dimensional characteristics of canonical TLS maturation stages, related to Figures 2 and 3

(A) Summary of 3D TLS classes identified in the reconstructed Patient1_B4 tissue image. 3D TLSs were categorized as Early, Primary, or Secondary based on CD20$^+$ B cells and CD21$^+$ and CD23$^+$ follicular dendritic cells (FDCs). Counts are shown for complete TLSs that passed QC, including subsets exhibiting Ki-67$^+$ germinal center (GC) cells, multifocal organization, and spatial association with granulomas.
(B) Per-section pseudoclassification versus true 3D TLS classification. Burnt orange dashed lines indicate true class proportions across 470 TLSs (see **Figure S4A**).
(C-D) Quantification of TLS size and volume across histological classes. Total cell count (B) and TLS volume (C) increase progressively from Early to Primary to Secondary TLSs, indicating coordinated structural expansion with maturation. Each point represents a single TLS.
(E) Comparative cell-type composition across TLS classes. Boxplots show relative proportions or absolute counts of major immune and non-immune populations. Secondary TLSs exhibit increased levels of total and Ki-67$^+$ B cells and PNAd$^+$ HEVs relative to Early and Primary TLSs. Proportionally, other cell types like CD4$^+$ and CD8$^+$ T cells, Tregs, CD11c$^+$ CD206$^+$ macrophages decline across maturation stages, reflecting relative contraction of these compartments as the B cell and follicular compartments expand. Center line, median; box, interquartile range; whiskers, 1.5× IQR. Top 5% of values are shown as jittered points; black crosses indicate means. Statistical significance was assessed by pairwise Mann-Whitney U tests ($P < 0.05$; *$P < 0.01$; **$P < 0.001$).
(F) Representative three-dimensional renderings of TLSs across maturation classes (early, primary, and secondary). Rows show marker groups highlighting B cell and follicular organization (CD20, CD21, CD23), T cell subsets (CD4, CD8a), proliferative activity (PCNA, Ki-67), vascular and stromal features (CD31, PNAd, αSMA), and myeloid populations (CD11c, CD206). These examples illustrate progressive follicular organization, immune compartmentalization, and proliferative activity with TLS maturation, while also highlighting intra-class heterogeneity. **Bottom:** Corresponding 2D projections of cellular point clouds for each TLS, generated by centering and principal axis alignment, with cells colored by phenotype to visualize spatial organization of B cells, T cells, and follicular domains.

bioRxiv preprint doi: https://doi.org/10.64898/2026.06.02.725201; this version posted June 18, 2026. The copyright holder for this preprint (which was not certified by peer review) is the author/funder, who has granted bioRxiv a license to display the preprint in perpetuity. It is made available under a CC-BY 4.0 International license.

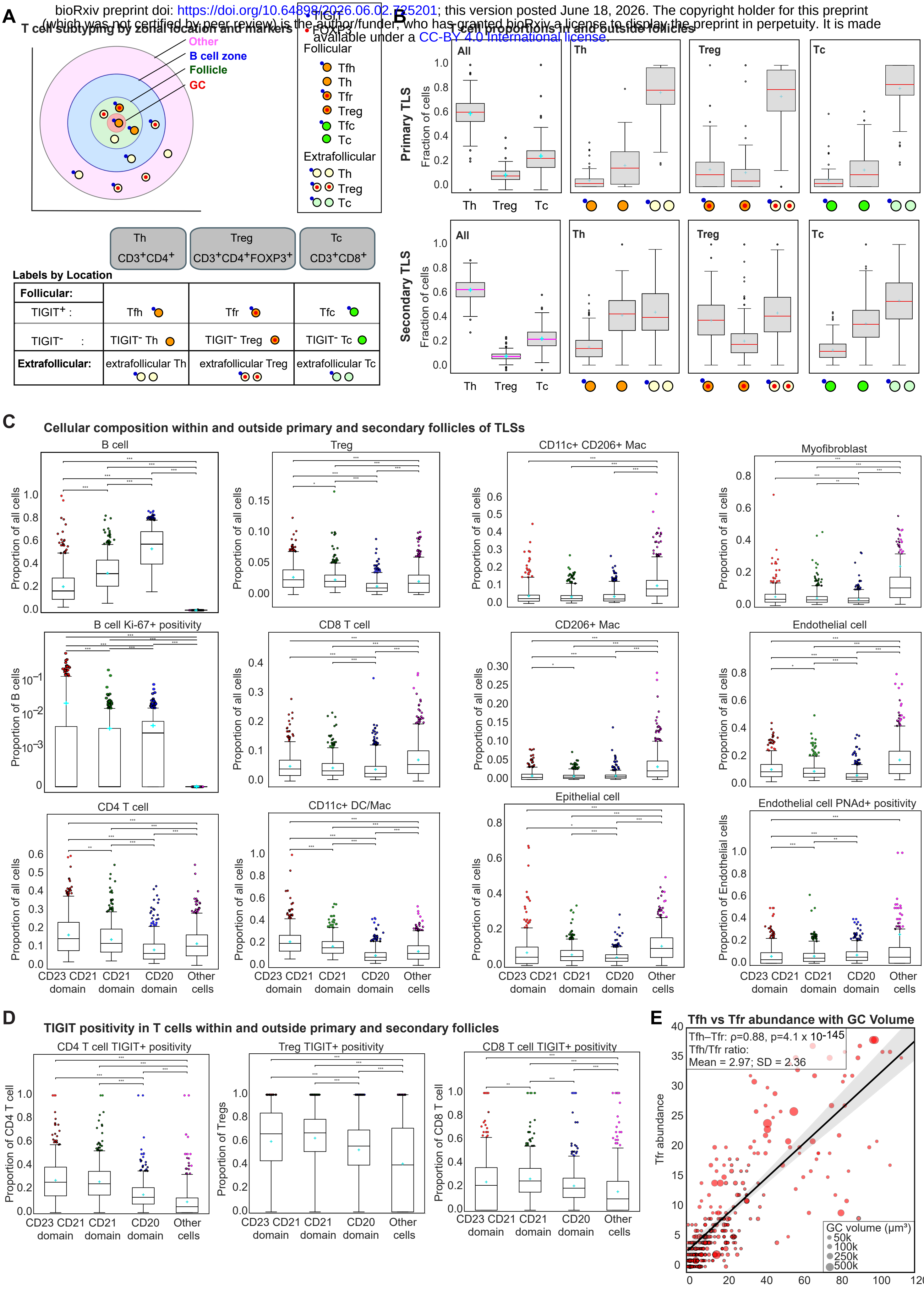

bioRxiv preprint doi: https://doi.org/10.64898/2026.06.02.725201; this version posted June 18, 2026. The copyright holder for this preprint (which was not certified by peer review) is the author/funder, who has granted bioRxiv a license to display the preprint in perpetuity. It is made available under a CC-BY 4.0 International license.

Figure S5. Spatial organization of immune cells across follicular and extrafollicular TLS compartments, related to Figure 3

(A) T cell subtyping by zonal location and markers. TLS schematic showing concentric shells based on mean volume-equivalent radii of TLS zones. The spatial positioning of follicular and extrafollicular T cells are also indicated. Also shown is the phenotyping scheme used to determine follicular and extrafollicular T cell states. We defined T follicular helper cells (Tfh) cells as CD3^{+} CD4^{+} TIGIT^{+} FOXP3^{-} in the follicle or GC; T follicular regulatory cells (Tfr) cells were similar but also FOXP3^{+}; and TIGIT^{+} CD3^{+} CD8^{+} T cells in GC or follicle were follicular cytotoxic T (Tfc) cells. TIGIT was used for quantification instead of PD1 because it had better signal to noise ratio. There were T cells in the follicle or GC that did not express TIGIT. T cells outside the follicle whether expressing TIGIT or not were defined as extrafollicular T helper (Th), Tregs, or cytotoxic T cells (Tc).

(B) Fractional composition of total and spatially-resolved T cell subsets within primary and secondary TLSs. First panels: fraction of CD4^{+} T cells, Tregs, and CD8^{+} T cells relative to their sum per TLS. 2nd-4th panels: fractional composition of spatially-resolved subsets of CD4^{+} T cells, Tregs, and CD8^{+} T cells. Boxplots show the distribution of per-TLS fractions, with cyan crosses representing means, center lines indicating medians, boxes representing interquartile ranges, whiskers denoting 1.5× IQR, and points representing individual TLSs.

(C) Quantification of cellular composition within and outside primary and secondary follicles. Boxplots show proportions of major immune, stromal, and structural cell types across CD20^{+}, CD21^{+}, CD23^{+}CD21^{+}, and “Other” regions, revealing strong zone-specific enrichment patterns. Center line, median; box limits, upper and lower quartiles, whiskers extend at most to 1.5x IQR; cyan crosses indicate means. Statistical comparisons were performed using pairwise Mann-Whitney U tests ($P < 0.05$; *$P < 0.01$; **$P < 0.001$).

(D) TIGIT positivity across T cell subsets stratified by spatial zones. Boxplots show the fraction of TIGIT^{+} cells among CD4^{+} T cells, Tregs, and CD8^{+} T cells within follicular (CD21^{+} and CD23^{+}CD21^{+}), CD20^{+}, and “Other” regions, demonstrating preferential enrichment of TIGIT^{+} T cells within follicular compartments.

(E) Tfh abundance positively correlates with Tfr abundance across CD21^{+}CD23^{+} GC domains. Here, abundance refers to cell counts. Spearman correlations: Tfh–Tfr, $\rho = 0.88$ ($p = 4.1 \times 10^{-145}$); Tfh:Tfr ratio–GC volume, $\rho = 0.05$ ($p = 0.36$). Mean Tfh:Tfr ratio = 2.97; SD = 2.36. Of the 435 GC domains, 338 (77.7%) contained both Tfh and Tfr cells and only these were

bioRxiv preprint doi: https://doi.org/10.64898/2026.06.02.725201; this version posted June 18, 2026. The copyright holder for this preprint (which was not certified by peer review) is the author/funder, who has granted bioRxiv a license to display the preprint in perpetuity. It is made available under a CC-BY 4.0 International license.

included in ratio analyses. Each point represents a GC domain and bubble size reflects 3D GC volume. Black line indicates linear regression with 95% confidence interval shading.

bioRxiv preprint doi: https://doi.org/10.64898/2026.06.02.725201; this version posted June 18, 2026. The copyright holder for this preprint (which was not certified by peer review) is the author/funder, who has granted bioRxiv a license to display the preprint in perpetuity. It is made available under a CC-BY 4.0 International license.

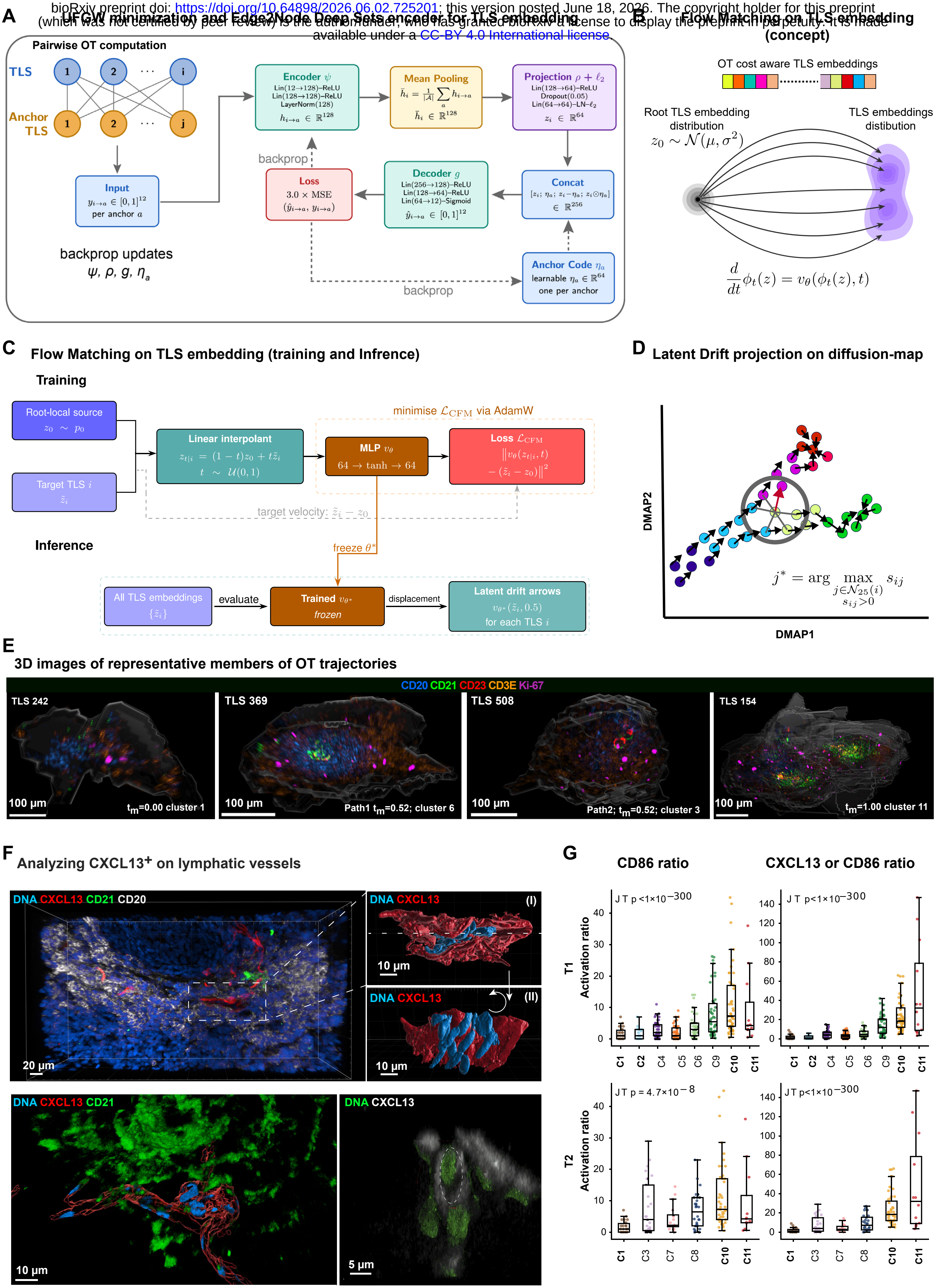

bioRxiv preprint doi: https://doi.org/10.64898/2026.06.02.725201; this version posted June 18, 2026. The copyright holder for this preprint (which was not certified by peer review) is the author/funder, who has granted bioRxiv a license to display the preprint in perpetuity. It is made available under a CC-BY 4.0 International license.

Figure S6. Maturation trajectory inference and follicular activation in TLSs, related to Figures 4 and 6

(A) UFGW minimization and Edge2Node Deep Sets encoder for TLS embedding. Pairwise transport-cost vectors between each TLS and a set of anchor TLSs were computed using Unbalanced Fused Gromov-Wasserstein (UFGW) minimization. UFGW jointly aligns the internal 3D spatial geometry of each TLS and penalizes phenotype mismatches across super-cells. It accommodates differences in total cell count via KL-penalized mass creation and removal terms. Each normalized edge vector $y_i \to a \in [0,1]^{12}$ encodes displacement, birth-cost, and death-cost across phenotypes. A shared encoder ψ maps each edge vector to a 128-dimensional hidden representation $h_i \to a$. These representations are aggregated by mean pooling over all anchors to yield a permutation-invariant node summary $h_i \in \mathbb{R}^{128}$. A projection head ρ followed by ℓ_2 normalization produces the final TLS embedding $z_i \in \mathbb{R}^{64}$. During training, a decoder g reconstructs each edge vector $\hat{y}_i \to a$ from z_i concatenated with a learnable anchor code η_a, minimizing a reconstruction loss via backpropagation through φ, ρ, g, and η_a. After training, z_i serves as the compact, permutation-invariant transport-cost embedding for each TLS, used in all downstream diffusion-map and trajectory analyses. For details, see supplementary method.

(B) Flow Matching on TLS embeddings (concept). OT-cost-aware TLS embeddings define a target distribution over the latent space (purple cloud, right). A root TLS source distribution $z_0 \sim \mathcal{N}(\mu, {\sigma_0}^2)$ is centered on the root TLS embedding (grey cloud, left). Flow matching learns a time-dependent vector field v_θ satisfying the probability flow ODE $\frac{d\phi_t(z)}{dt} = v_\theta(\phi_t(z), t)$, which continuously transports probability mass from the root distribution toward the target TLS distribution, representing a directed developmental drift across the latent space.

(C) Flow Matching training and inference pipeline. A flow matching model is trained to learn a latent vector field v_θ that maps each TLS embedding toward its most probable next state. To this end, for every TLS_i a source point $z_0 \sim \mathcal{N}(\mu, {\sigma_0}^2)$ is sampled and a linear interpolant $z_{t|i} = (1-t)z_0 + t\tilde{z}_i$ is constructed at uniformly sampled $z_0 \sim \mathcal{U}(0,1)$. A time-conditioned MLP is trained to predict the conditional velocity $\tilde{z}_i - z_0$ along the probability path, minimizing $\mathcal{L}_{CFM}$ via AdamW for 10,000 epochs. Once trained, the model is used to infer displacement vector indicating the next most probable state in the latent space (Latent drift arrows). For details, see Supplementary Methods

(D) Latent drift projection onto the diffusion map manifold and identifying the next most probable TLS state. The latent drift vector is projected onto the diffusion-map manifold using the Nyström

bioRxiv preprint doi: https://doi.org/10.64898/2026.06.02.725201; this version posted June 18, 2026. The copyright holder for this preprint (which was not certified by peer review) is the author/funder, who has granted bioRxiv a license to display the preprint in perpetuity. It is made available under a CC-BY 4.0 International license.

extension approach (see Supplementary Information). The resulting displacement is then used to calculate an alignment score $S_{ij} = u_i \cdot d_{ij}$ for each of the k = 25 nearest neighbors in DMAP space. The neighbor with the highest positive alignment score, $j^* = \underset{j \in \mathcal{N}_{25}(i),\, s_{ij>0}}{\operatorname{argmax}} s_{ij}$, identifies the most probable next developmental state and defines the direction arrow visualized on the diffusion map manifold.

(E) Representative 3D renderings of TLSs sampled along T1 and T2 at comparable flow times: TLS 242 (t_m = 0.00), TLS 369 (t_m = 0.52 on T1), TLS 508 (t_m = 0.52 on T2), and TLS 154 (t_m = 1.00).

(F) $CXCL13^+$ vessel-like structures show nuclear morphologies consistent with lymphatic endothelial vessels. Top left panel: Volumetric rendering of a TLS imaged with 3D CyCIF (DNA (blue), CXCL13 (red), CD21 (green), CD20 (white)). Scale bar is 20 μm. Top right panel (I): Zoomed-in view of a $CXCL13^+$ vessel from top left panel (dashed box) and represented by surface rendering. Channels shown are DNA (blue) and CXCL13 (red). Scale bar is 10 μm. Top right panel (II): Cutaway section along dashed line in middle top panel showing multiple elongated nuclei (blue) within $CXCL13^+$ vessel. Scale bar is 10 μm. Bottom left panel: Volumetric rendering of a different TLS (DNA (blue), CXCL13 (red), CD21 (green). $CXCL13^+$ vessel is represented as translucent surface rendering (red). Nuclei (blue) shown are in contact with inner wall of vessel. Scale bar is 10 μm.

Bottom right panel: Cross section of a 3D CyCIF dataset showing Hoechst-stained nuclei (green) within CXCL13 vessel (grey). Dashed outline highlights a vascular lumen containing nuclei. Scale bar is 5 microns.

(G) Activated FDC ratios relative to inactive FDCs increase across clusters along both paths. Activation ratios are shown for $CD86^+$ only (left) and $CXCL13^+$ or $CD86^+$ FDCs (right). FDCs are defined as ($CD21^+$ or $CD23^+$) $CD20^-$ $CD3E^-$ cells. Left: ratio of $CD86^+$ FDCs to (1 + $CD86^-$ FDCs). Right: ratio of ($CXCL13^+$ or $CD86^+$) FDCs to (1 + $CXCL13^-$ $CD86^-$ FDCs). p-values are from the Jonckheere-Terpstra trend test.

bioRxiv preprint doi: https://doi.org/10.64898/2026.06.03.725201; this version posted June 18, 2026. The copyright holder for this preprint (which was not certified by peer review) is the author/funder, who has granted bioRxiv a license to display the preprint in perpetuity. It is made available under a CC-BY 4.0 International license.

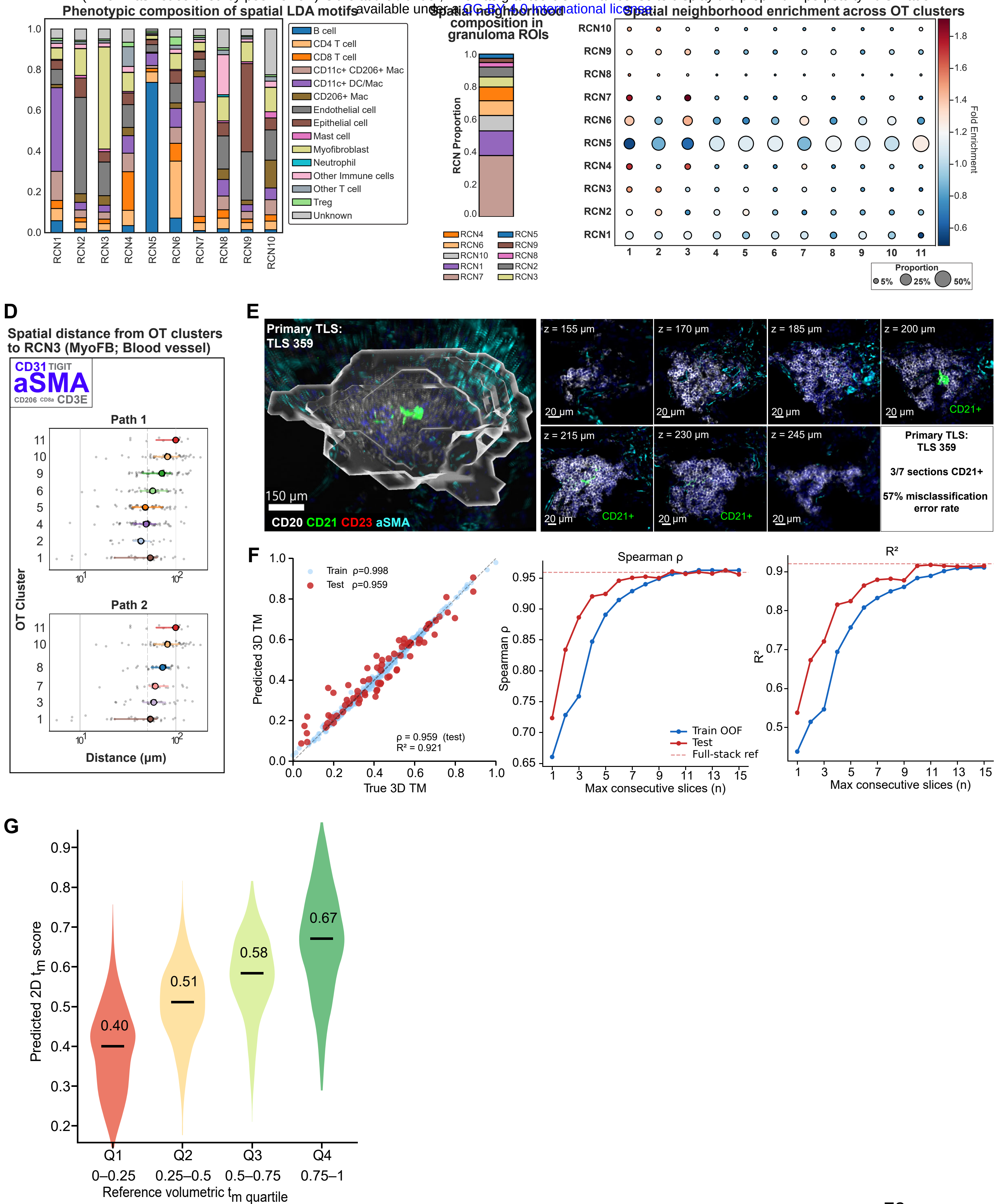

bioRxiv preprint doi: https://doi.org/10.64898/2026.06.02.725201; this version posted June 18, 2026. The copyright holder for this preprint (which was not certified by peer review) is the author/funder, who has granted bioRxiv a license to display the preprint in perpetuity. It is made available under a CC-BY 4.0 International license.

Figure S7. Spatial motif architecture and prediction of 3D TLS maturation state from 2D tissue sections, related to Figure 7

(A) Phenotypic composition of spatial motifs identified by latent Dirichlet allocation (LDA), representing recurrent cellular neighborhoods.
(B) Motif composition within granuloma regions-of-interest (ROIs).
(C) Motif enrichment across TLS OT clusters, with bubble size indicating motif proportion and color representing relative enrichment.
(D) Distance from TLS OT clusters to RCN3 which corresponded to a $CD31^+$ αSMA^+ neighborhood.
(E) Example of sectioning-based misclassification in a primary TLS. Individual serial sections variably display CD21 positivity, leading to apparent class switching in 2D.
(F) Prediction of 3D TLS maturity (t_m; mass-normalized time) from consecutive 2D sections using a gradient boosting model. Left: predicted versus true t_m (train ρ = 0.998; test ρ = 0.959). Center/right: performance (Spearman ρ and R^2) improves with increasing slice number, approaching full-stack accuracy at ~11–13 sections.
(G) Prediction of 3D TLS maturity from single 2D TLS sections. Predicted 2D t_m scores stratified by reference 3D t_m quartiles (Q1-Q4), showing a monotonic increase in predicted maturity. Prediction accuracy was highest for TLSs with intermediate reference 3D t_m values (0.25–0.75), whereas highly mature TLSs showed greater variability, consistent with increased structural heterogeneity. See also **Figure 7H**.
